# Case Report: The Value of Genomic Analysis in a Case of Megakaryoblastic Leukemia With Atypical Initial Manifestation

**DOI:** 10.3389/fped.2022.875510

**Published:** 2022-06-29

**Authors:** Miriam Gutiérrez-Jimeno, Elena Panizo-Morgado, Marta Calvo-Imirizaldu, Víctor Galán-Gómez, Adela Escudero-López, Ana Patiño-García

**Affiliations:** ^1^Department of Pediatrics, University Clinic of Navarra, Pamplona, Spain; ^2^Division of Neuroradiology, Department of Radiology, University Clinic of Navarra, Pamplona, Spain; ^3^Pediatric Hematology and Oncology Unit, Department of Pediatrics, La Paz University Hospital, Madrid, Spain; ^4^Pediatric Molecular Hemato-Oncology Section, Department of Genetics, Institute of Medical and Molecular Genetics (INGEMM), La Paz University Hospital–IdiPAZ, Madrid, Spain; ^5^Solid Tumor Program, CIMA, Center for Applied Medical Research and IdiSNA, Pamplona, Spain

**Keywords:** extramedullary acute myeloid leukemia, myeloid sarcoma, acute megakaryoblastic leukemia, genomics, next generation sequencing

## Abstract

We report the case of a 7-month-old female patient who developed acute megakaryoblastic leukemia 6 months after the appearance of skull bone lesions. Initial evaluation and diagnosis of this patient were challenging and only achieved thanks to genomic analysis by NGS (next generation sequencing). It is unusual for the initial manifestation of acute megakaryoblastic leukemia to be a skull bone lesion. Extramedullary acute myeloid leukemia (eAML), also known as myeloid sarcoma (MS), often occurs simultaneously with acute myeloid leukemia (AML), although it may precede AML. Genomic analysis based on a NGS panel (Oncomine Childhood Cancer Research Assay) detected a *RBM15::MKL1* fusion, a consequence of a *t* (1;22)(p13;q13) translocation, establishing the diagnosis of acute megakaryoblastic leukemia and enabling disease follow-up by qPCR. A diagnosis of eAML is built up from various findings in radiological, histological, immunophenotypic and genomic studies; when the tumor appears *de novo*, diagnosis is more complicated. We emphasize the importance of a multidisciplinary team in the initial approach to rare tumors and the use of genomic studies to contribute to the knowledge of these neoplasms, risk stratification and treatment planning.

## Introduction

The World Health Organization (WHO) defines an extramedullary acute myeloid leukemia (eAML) tumor as a tumoral mass consisting of myeloid blasts occurring at an anatomic site other than bone marrow. eAML may also precede (in 25% of cases) or follow (in most cases) the development of an acute myeloid leukemia, a myeloproliferative neoplasm, or a myelodysplastic syndrome (1). The incidence of eAML is higher in children than in adults (2) and is associated with age of <1 year, central nervous system (CNS) disease, AML type M5 according to FAB classification, abnormal cytogenetics and poor clinical outcome (3). Although eAML has been the subject of more than 2,000 case reports in the medical literature, there are <50 reported cases of eAML with megakaryoblastic differentiation, which reflects the rarity of the neoplasm and the difficulty of diagnosis (4). Many misdiagnoses have been documented (5).

## Case Description

A 7-month-old infant girl, of African descent, who was under neuropediatric follow-up after premature birth, was evaluated at her referral center because of the appearance of a frontal tumor (June 2020). There was no history of previous trauma and nothing of relevance in the family's medical records. Physical examination found multiple congenital dermal melanocytosis lesions and macrocephaly (p>99, 3.66 SD). Ultrasound (US) study of the frontal tumor showed a nodular lesion not suggestive of malignancy, and therefore it was decided to follow the lesion closely. Three months later a skull X-ray revealed an osteolytic lesion in the right frontal region. Subsequent cranial MRI (Magnetic Resonance Imaging) revealed destructive/infiltrative bone lesions in the right frontal region, left parietal and left external sphenoid area, with a component of epicranial and intracranial soft tissue involvement with apparent disruption of the dura mater. There was also possibly an intradural component.

The case was referred to a specialized center in pediatric oncology where a bone biopsy was performed in October 2020. At that time, the lesion was thought most likely to be a solid tumor, such as, neuroblastoma or sarcoma. Differential diagnosis was made with eAML in the context of myeloproliferative disease and the more unlikely histiocytosis. The pathological study of the biopsy from the bone lesion found small round and partially-blue cells with clear chromatin, an evident nucleus and artifacts. Immunocytochemical study found positivity for vimentin and INI1, CD68 +/-, and negativity for synaptophysin, SC-100, CD56, neurofilaments, actin, desmin, CK AE1/AE3, EMA, CD34, CD99, PLAP, CD30, CD45 and WT1. The Ki-67 proliferation index was high. Vimentin is a nonspecific marker; however, a tumor with negative vimentin is unlikely to be a sarcoma, a lymphoma or a melanoma; being positive, it was first suspected to be a solid tumor (e.g., a sarcoma). In the case of INI, loss of *SMARCB1* (INI1) expression is identified in a variety of rhabdoid and non-rhabdoid CNS neoplasms and in epithelioid sarcoma and is also a rather unspecific marker. *SMARCB1* was subsequently discarded in the NGS analysis. The pathologic diagnosis was undifferentiated infantile malignant round cell neoplasm. Thoracic-abdominal CT found no evidence of metastatic disease.

**Figure 1:**
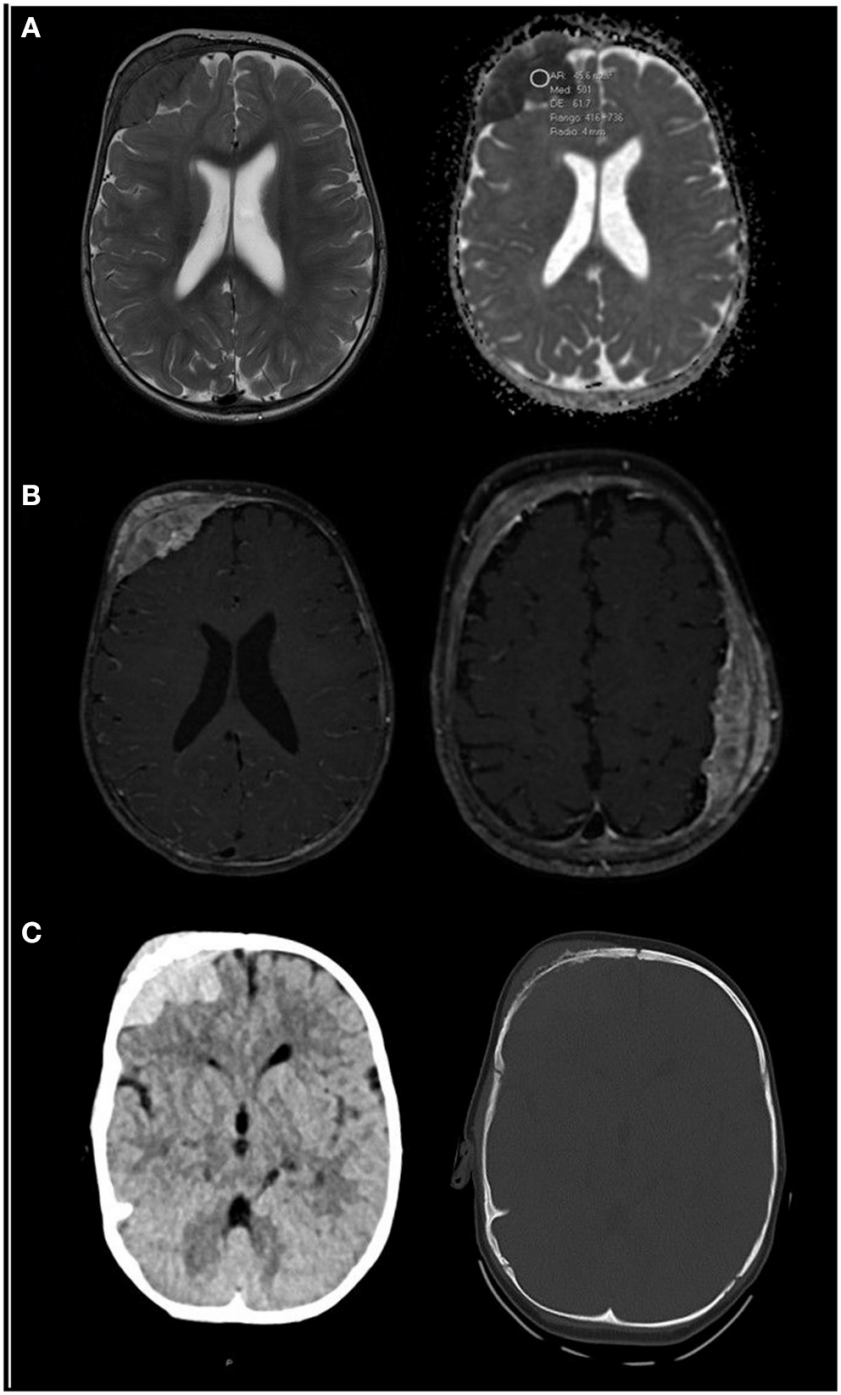
**(A)** Axial T2-weighted (right image) and apparent diffusion coefficient (ADC, left image) MRI sequences. A right frontal soft tissue mass is patent, hypo-intense on T2 and with low ADC values (500), which likely represent high cellularity. **(B)** Axial contrast-enhanced T1-weighted images with fat saturation at two levels show bilateral masses, right frontal and left parietal, with homogeneous enhancement. **(C)** Unenhanced CT scan centered in the right frontal mass shows spontaneous hyper-density in the parenchymal window (right image), also typical of hyper-cellular tumors. Bone window (left image) shows erosive changes within the subjacent bone. Findings are consistent with myeloid sarcoma. MRI scans were taken in December 2020.

In December 2020, the patient was admitted to our center for a second opinion. The infant then had anemia (10,7 g/dL) and thrombocytopenia (62 x 10^9^/L). Leukocytes were in normal range, 9.28 x 10^9^/L (6-15 x 10^9^/L). Peripheral blood morphology: intense anisopoikilocytosis of red series with hypochromia; occasional neutrophils with mirror nuclei and clumping, while others well granulated and segmented. Frequent stimulated lymphocytes without blasts. Thrombocytopenia with anisothrombia and some large platelets. Bicytopenia without blasts with morphologic alterations in the two series: mild in the granulocytic series and moderate in the red and platelet series. The findings of additional MRI and computerized tomography (CT) scans were consistent with myeloid sarcoma (Figure 1). Bone marrow aspirate and biopsy were performed in the left iliac crest, showing presence of interstitial blast-like cells, negative for CD34 and CD117, and bone marrow aspirate in the right tibia. Cells of the three hematopoietic lineages were underrepresented and there were clusters of cells, with blue cytoplasm, that appeared to be infiltrated by a solid tumor. Aspirate samples were studied by two different pathology centers. One center reported bone marrow with excess of blasts but <10% positive for CD41 with presence of medium-sized cells with a tendency to coalesce, compatible with metastases probably of non-hematologic lineage. The other center reported bone marrow with presence of numerous interstitial blast-like cells, of intermediate size, with open chromatin and inconspicuous nucleoli, sometimes multiple (the overall cellularity was approximately 75%). Bone marrow cytometry shows the presence of 3% of cells with the following phenotype: CD34-, CD117(l)+, HLADR-CD41+, CD42a+, CD42b+, CD61+, CD9+, CD13(lo)+, CD36(lo)+, CD33-, CD64-, CD300e-, CD14-, CD16-, CDIlb-, CD10-, CD15-, CD11c-, CD123-, CD105-, CD25-, CD203c-, NG2-, CD3-, CD3cyto-, CD4-, CD2-, CD8-, CD5-, CDIa-, CD56-, TaT-, CD19-, CD22-, CD22cyto-, MPO cyto-, Lysozyme-, CD45verylo+, with abnormal expression of CD7. The study by immunohistochemistry was negative for CD34 and CD117, and positive for CD31, MPO and CD15, with cells that phenotypically corresponded to myeloid precursors. One of the likely explanations for CD45 being negative and then positive is that as the disease progresses, the cell clones become easier to characterize.

Given the discordance between the previous diagnosis of small round blue cell tumor and the new findings, and after consideration of the case by our hospital's tumor board, a genomic study was requested. NGS analysis with the Oncomine Childhood Research Assay (Thermo Fischer) was performed both in the bone marrow with a 4% tumor infiltration and a biopsy provided from the referring center with unknown tumor percentage. Both analyses identified the presence of a fusion between exon 1 of the *RBM15* gene and exon 4 of the *MKL1* gene (149,313 reads in the tumor biopsy specimen, 9,165 reads in the infiltrated bone marrow specimen). This fusion gene is a consequence of a *t* (1;22)(p13;q13) translocation, a genetic alteration associated with acute megakaryoblastic leukemia.

**Figure 2:**
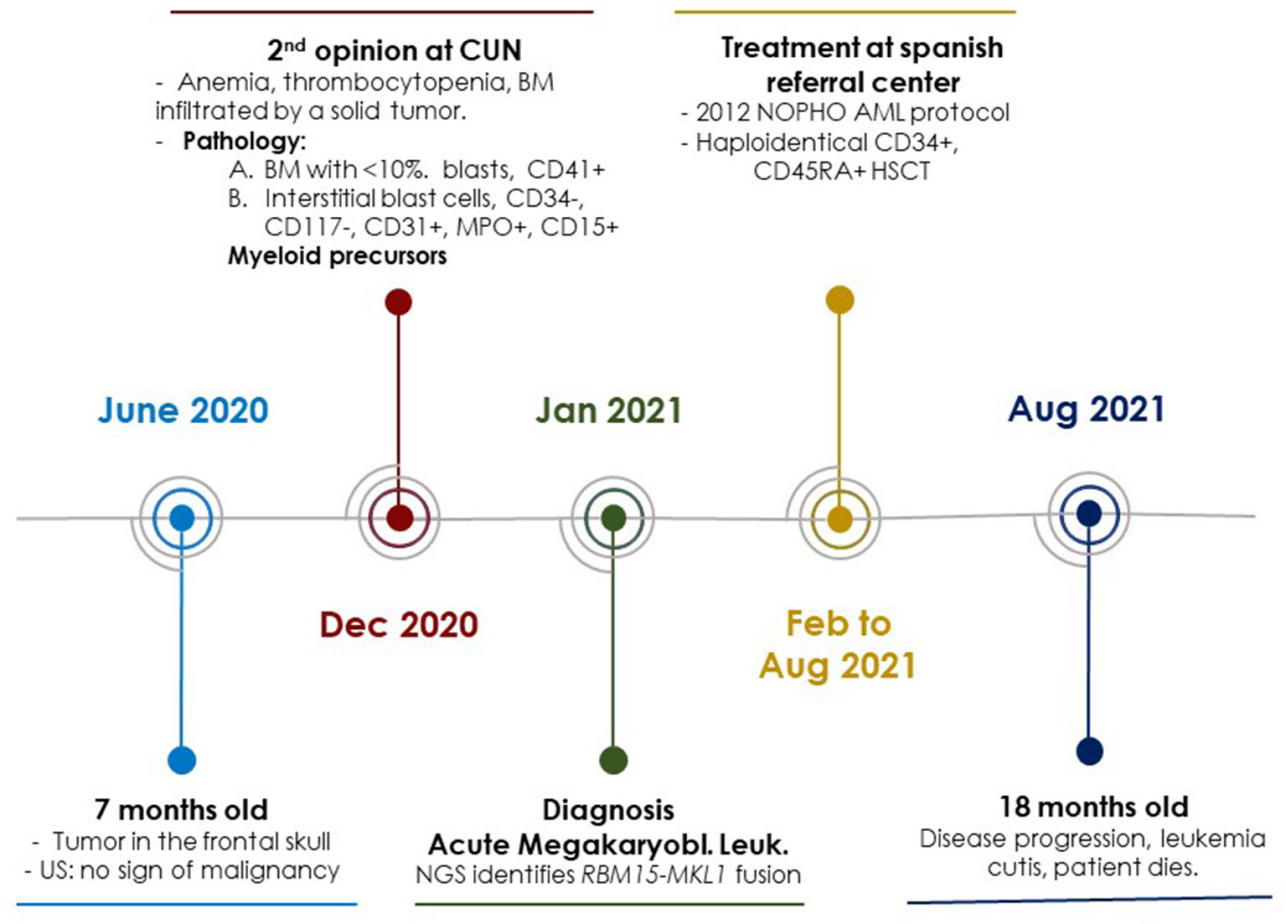
Schematic representation of the most important events in the diagnosis, treatment and follow-up of the clinical case. CUN, Clínica Universidad de Navarra; US, Ultrasound Study; BM, Bone Marrow; NOPHO, Nordic Society of Pediatric Hematology and Oncology; AML, Acute Myeloid Leukemia; NGS, Next Generation Sequencing.

The patient was transferred to the referral Spanish center in January 2021 (Hospital La Paz, Madrid, Spain), where bone marrow biopsy was repeated, and immunohistochemistry confirmed the cell population to be CD61 positive. Treatment was initiated 1 week after diagnosis according to the 2012 NOPHO acute myeloblastic leukemia protocol: two induction cycles plus one cycle of consolidation followed by skull radiotherapy (24 Gy administered in 12 fractions for persistence of extramedullary disease) were given. In July 2021, at the end of the consolidation cycle, bone marrow biopsy showed measurable disease, a population of: CD117+, CD34-, CD45+m, CD41+, CD36-, CD123+d-, CD9-/+, CD38+m phenotype corresponding to megakaryocytic precursors, very few CD34+ myeloid precursors (0.03%), scarce granulocytic maturation, with undetectable monocytes and very scarce erythroid series. These findings compatible with refractory MLA of megakaryoblastic component. In this point, 8 months after diagnosis, two courses of salvage chemotherapy were given and haplo-identical CD45RA+ lymphocyte-depleted hematopoietic stem cell transplantation (HSCT) was performed in aplasia without blast status. It is a fact of interest that between the two salvage cycles, the disease progressed and a diagnosis of leukemia cutis was confirmed by biopsy. The skin lesions evolved to hyperpigmented residual lesions with treatment, but the bone lesions persisted without significant reduction despite the intensive treatment regimen (Figure 2).

During all the stages of disease follow-up, it was difficult to obtain bone marrow aspirates, due to the patient's constitution and age, and corresponding biopsies were complex to interpret, largely because of fibrosis, as well as post-chemotherapy changes and aplasia.

**Figure 3:**
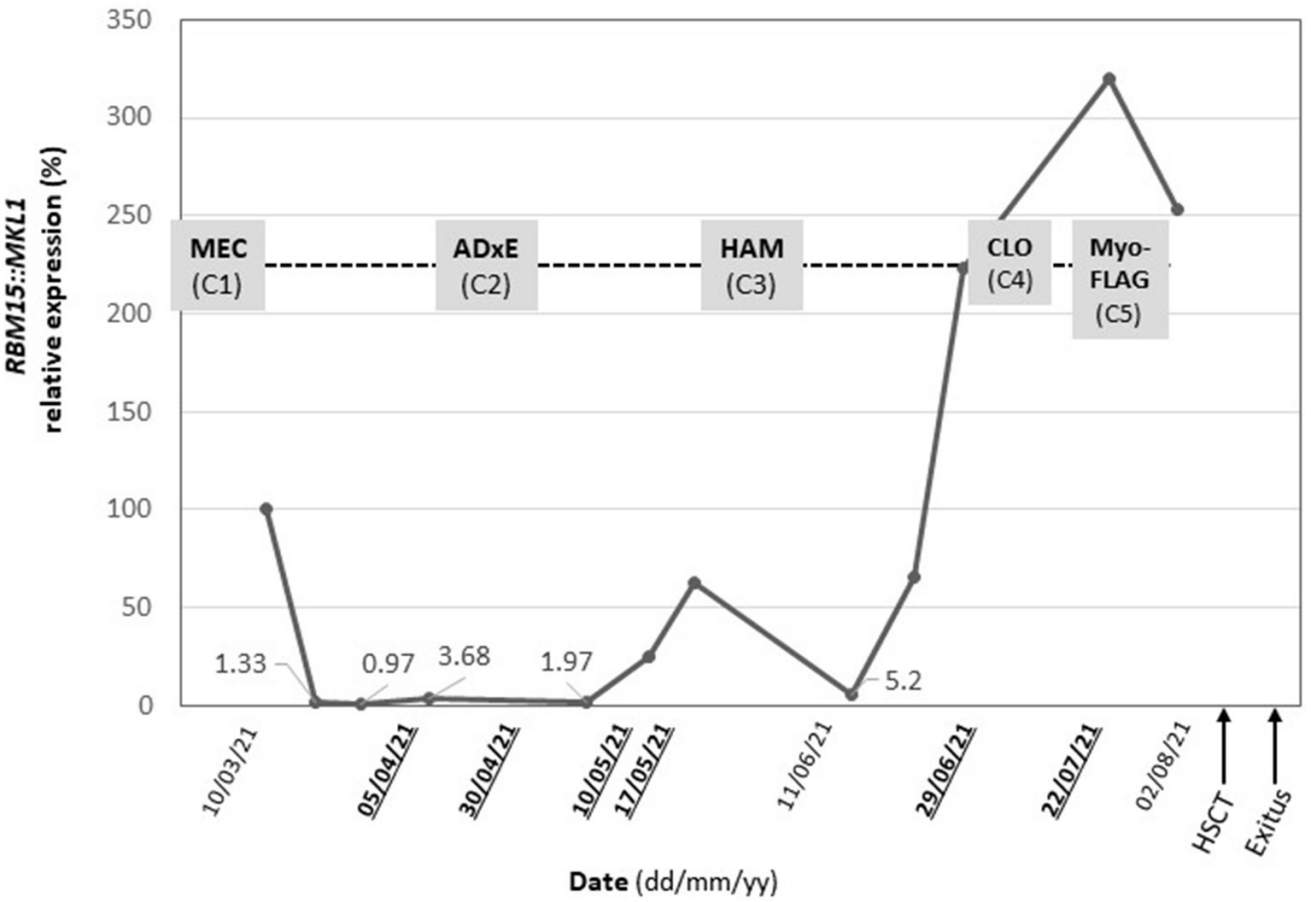
Follow-up of minimal residual disease (black) by qPCR and treatment plan (grey). *RBM15::MKL1* relative expression and administered chemotherapy following the NOPHO-DBH AML 2012 Dates highlighted (bold underlined) indicate data points in which multiparametric flow cytometry analyses were evaluable. Protocol from diagnosis to patient death. MEC, (mitoxantrone in first course) and ADxE (cytarabine, liposomal daunorubicin, etoposide): induction course, HAM (High-dose cytosine arabinoside and mitoxantrone): consolidation course, CLO (clofarabine) and FLA (fludarabine): salvage therapy, HSCT, Hematopoietic stem cell transplantation.

Minimal residual disease and pathology studies were performed in parallel throughout the patient's treatment (at diagnosis, during induction chemotherapy, during consolidation treatment, at diagnosis of refractory disease, and during salvage chemotherapy) by flow cytometry and biopsy of bone marrow respectively. Minimal residual disease was measured by *RBM15::MKL1*fusion gene detection using qPCR (LC480 System (Roche). The tumor fusion gene was detectable at all follow up points, always by performing a bone marrow biopsy (Figure 3). Immunophenotyping and MRD monitoring by multiparametric flow cytometry and imaging indicated persistence of extramedullary disease in all time points evaluable. Many aspirate samples in this patient were not evaluable and samples were extremely difficult to obtain and analyze, so that only limited time points were available and positive for extramedullary disease (Figure 3).

In august 2021, at 18 months old, the patient died due to disease progression.

## Discussion

eAML is composed of immature cells of the granulocytic series, originating from a myeloid precursor^1^, it might also originate from monocytic, megakaryocytic, and erythroid lineage. Megakaryocytic differentiation is extremely rare (6). When eAML precedes the development of AML or when it develops into a subtype of AML, it can be extremely difficult to arrive at a diagnosis of acute megakaryoblastic leukemia, which often presents with severe myelofibrosis (7). However, although it is a characteristic finding of this type of disease, in our patient, perhaps because she was a child, it was not significantly documented.

For our patient, it took 3 months to establish a definitive diagnosis. The initial hypothesis was that the bone lesion was a solid tumor or metastasis, probably of non-hematological origin. After repeating the tests and referral to expert pathologists in pediatric tumors, the initial hypothesis was disregarded, but it was not until the NGS genomic study was performed that the definitive diagnosis of acute megakaryoblastic leukemia was reached. The pathological discordance encountered can be attributed to the fact that, in this patient, it was always difficult to obtain and interpret bone marrow aspirates. In any event, genomic analysis is arguably necessary for detection of this or any other solid tumor with a pathognomonic genomic feature, whether it be mutation or rearrangement. Therefore, this case highlights the need for multidisciplinary teams and the use of tests complementary to conventional histology and immunohistochemistry.

In addition, when extramedullary disease appears in *de novo* eAML, the extramedullary disease can be confused with other tumors, which has led to misdiagnosis in 40% of the cases reported in the literature (8, 9). Most patients with *de novo* eAML develop AML within 4 to 12 months^5^, and consequently it is imperative to reduce the time taken for diagnosis so that chemotherapy treatment can be started early (induction treatment), and optimally, as soon as the presence of eAML appears and/or is suspected.

Clinical manifestations such as those reported herein should alert physicians to take, without delay, all necessary measures to differentiate between the most likely diagnoses: AML, non-Hodgkin lymphoma, histiocytosis, neuroblastoma, sarcomas, and blue round cell tumors. These measures should include tumor and bone marrow biopsy, imaging tests (MRI, CT, PET), histological studies, immunohistochemistry studies, immunophenotyping and MRD monitoring by multiparametric flow cytometry and NGS genomic studies designed for a complete genomic profile of cancers affecting children and young adults. If it is difficult to obtain and/or interpret bone marrow biopsies, molecular testing (quantitative PCR) is key to monitoring disease progression and response to treatment (10). In fact, in the case we report here, none of the cytometry/cytology evaluations were valid, and the only feasible technique for follow-up was qPCR.

eAML seems to be more frequent in children than in adults, and so pediatric oncologists need be aware of the clinical and biological hallmarks in order to diagnose and stratify this group of tumors. Megakaryoblastic differentiation of eAML, affects multiple locations, rarely presents *de novo* and might have a worse prognosis (4). In general, the most frequently affected locations are subcutaneous tissue and the orbit. In our patient, osteolytic bone lesions preceded leukemia, and cutaneous lesions (leukemia cutis) occurred during tumor progression, ~1 month before the patient's death. Unlike the lesion in the calvaria, the skin disease almost disappeared with salvage chemotherapy, only leaving residual hyperpigmentation.

Immunohistochemical panels including CD43, MPO, CD117, CD68, CD3 and CD20 might successfully identify eAML in most cases, provided there is no difficulty in obtaining the sample. In cases with megakaryoblastic and/or erythroblastic differentiation, FVIII, CD41, CD61, glycophorin A and hemoglobin A should be included on the diagnostic panel (4). In our case, a bone marrow biopsy was performed and informed with an extensive interstitial infiltration (70%) of CD61-positive blasts, suggesting an acute myeloid leukemia (megakarioblastic). During follow up, CD61 expression without co-expression of FVIII was evaluated.

The prognosis for patients with eAML is variable and depends on several factors, such as cytogenetic risk profile, whether involvement of bone marrow is isolated or concurrent (*de novo*, with simultaneous AML, myelodysplastic syndromes, or myeloproliferative disorder) (4), response to induction therapy and the presence of any addressable mutations at the extramedullary site or in marrow samples. In acute megakaryoblastic leukemia, *NUP98::KDM5A, CBFA2T3::GLIS2, KMT2A* rearrangements and monosomy 7 have been shown to be associated with poor outcome while *RBM15::MKL1* and other alterations seem to be associated with a better prognosis (11, 12). Nevertheless, as stated before, outcomes with eAML are in general poor, even in cases, such as that reported here, in which the genetic aberration is *a priori* associated with a better prognosis. Prognostic categorization of genetic aberrations should take into account the clinical entity in which they manifest.

In conclusion, there is a high rate of misdiagnosis of eAML, but diagnosis is important in order to establish the approach to therapy. If the differentiation is megakaryocytic, diagnosis can be especially difficult because it can be difficult to obtain and study samples with conventional methods. Advanced diagnostic techniques are required, and, furthermore, these represent our best hope for learning more about this rare disease. Genomic studies based on NGS promise not only to be the cornerstone of diagnosis but also to allow us to continue improving management of the disease by opening up the possibility of using genomic findings to target therapy and by inclusion in clinical trials.

## Data Availability Statement

The datasets for this article are not publicly available due to concerns regarding participant/patient anonymity. Requests to access the datasets should be directed to the corresponding author.

## Ethics Statement

The studies involving human participants were reviewed and approved by Ethics Committee of Clínica Universidad de Navarra. Written informed consent to participate in this study was provided by the participants' legal guardian/next of kin. Written informed consent was obtained from the individual(s) for the publication of any potentially identifiable images or data included in this article.

## Author Contributions

MG-J was responsible for gathering the clinical data and contributed to the genetic analysis under supervision of AP-G. EP-M and VG-G were responsible for patient treatment, clinical data, family communication, obtaining samples, and informed consent issues. MC-I was responsible for the imaging study and performed on the patient and collaborated in the drafting of the manuscript. VG-G and AE-L oversaw following up the patient with genomic studies and made the graphs provided in the manuscript. AP-G was the coordinator of the team and direct responsible of genetic analyses. All authors contributed to the article and approved the submitted version.

## Funding

Fundación La Caixa grant (P-GA), Gobierno de Navarra, Department of Health (P-GA).

## Conflict of Interest

The authors declare that the research was conducted in the absence of any commercial or financial relationships that could be construed as a potential conflict of interest.

## Publisher's Note

All claims expressed in this article are solely those of the authors and do not necessarily represent those of their affiliated organizations, or those of the publisher, the editors and the reviewers. Any product that may be evaluated in this article, or claim that may be made by its manufacturer, is not guaranteed or endorsed by the publisher.
